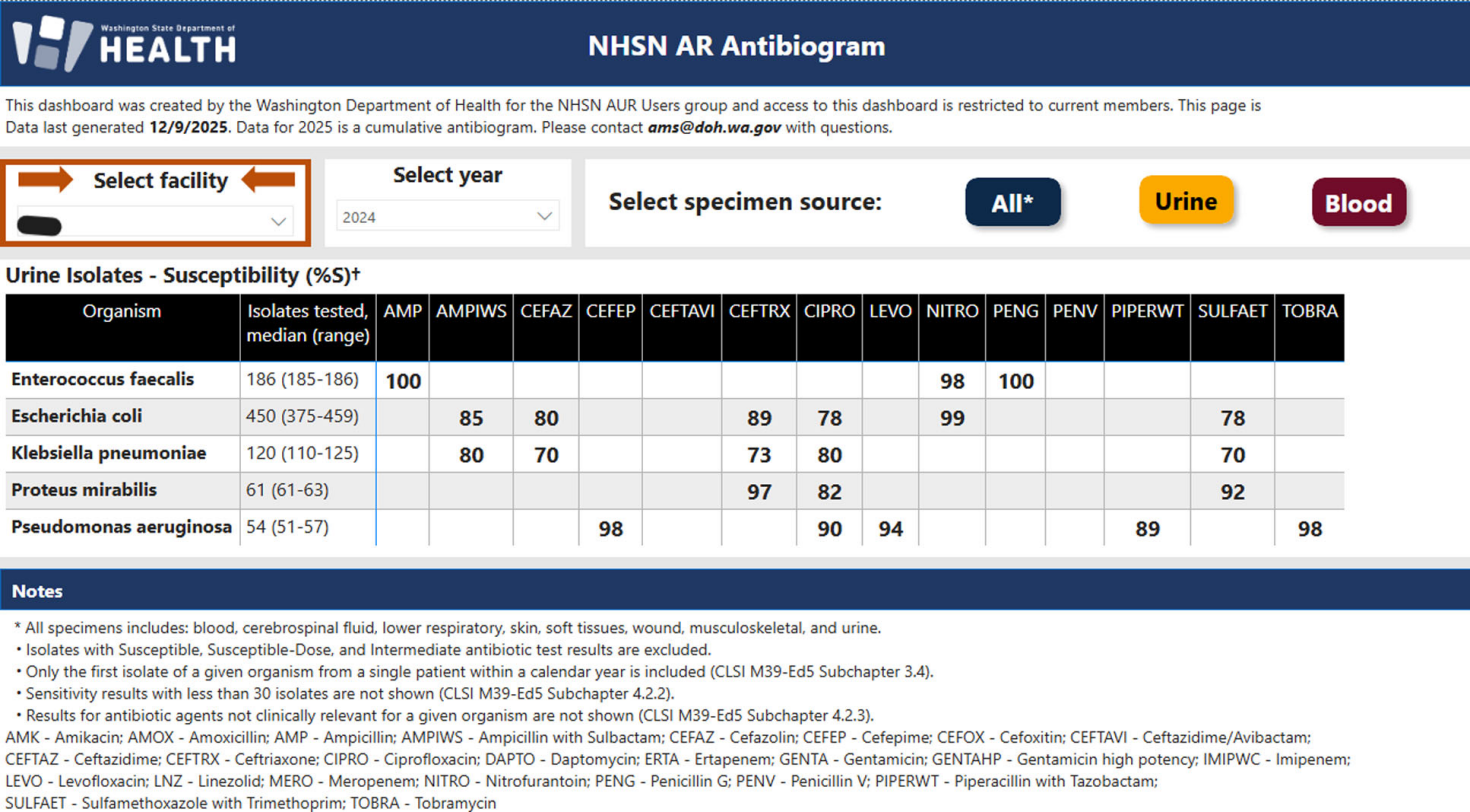# 100 A Model for High Reliability in Infection Prevention: The Role of the Generalist, Specialist and Centralized Surveillance in Infection

**DOI:** 10.1017/ash.2026.10434

**Published:** 2026-06-23

**Authors:** Gabriel Eligado, Jessica Zering, Erica Stohs, Kamenar Katarina

**Affiliations:** 1 Washington State Department of Health; 2 Creighton University Medical Center

## Abstract

**Background:** The National Healthcare Safety Network Antimicrobial Resistance Option (NHSN AR) is the nation’s primary tracking system for antimicrobial resistance. As of December 2025, 89% of Washington (WA) acute care hospitals are reporting antimicrobial susceptibility data to the AR option. The AR option provides a feature to generate antibiograms, a clinical tool to guide empiric antibiotic selection. However, the NHSN generated antibiograms do not adhere to the Clinical and Laboratory Standards Institute’s widely used M39 (CLSI M39) guidelines, which are best practices for antibiogram creation. Due to concerns of limited clinical applicability, we sought to create a CLSIM39 compliant antibiogram dashboard using NHSN AR data to support hospitals to make informed clinical decisions and track local resistance data. **Methods:** We downloaded the line list for All Antimicrobial Resistance Events from NHSN, transformed the data in R, and imported it into PowerBI to generate interactive antibiogram reports. The initial dashboard focused on the most commonly reported Gram-negative pathogens in WA: Enterococcus faecalis, Escherichia coli, Klebsiella pneumoniae, Proteus mirabilis, and Pseudomonas aeruginosa. We included isolates from blood, cerebrospinal fluid, lower respiratory tract, skin and soft tissue, wound, musculoskeletal, and urine specimens. Following CLSI M39 guidelines, we retained only the first isolate per organism per patient per calendar year. For specimen-specific reporting, we applied specimen filters by source prior to isolate selection. We selected antimicrobials and final susceptibility interpretations to calculate percent susceptible based on CLSIM39 and M100 guidelines. We suppressed organism-agent combinations with fewer than 30 isolates tested or lack of clinical relevance. To improve interpretability, we included the median and range of isolates tested for each organism. Three antibiograms were available: all-specimen sources, blood only, and urine only. To ensure data security, we assigned hospital alias codes and hosted the dashboard on a secure PowerBI platform. **Results:** WA DOH launched the NHSN AR Antibiogram Dashboard in December 2025. The dashboard displays susceptibility data based on analysis of 209,836 isolates collected between 1/1/2020 and 11/30/2025. We generated 74 all-specimen, 43 blood, and 74 urine-specific facility antibiograms. Currently, 93 antimicrobial stewards representing 80 hospitals have dashboard access, enabling them to track antimicrobial resistance trends year-to-year and compare resistance patterns by specimen source. **Conclusion:** The dashboard equips antimicrobial stewards with actionable data to monitor local resistance trends and inform facility-specific antimicrobial use guidelines. We plan to expand the dashboard to include additional organisms and specimen-specific reports.

**Figure:**